# Unveiling the Catalytic Pathway of Rh(II)/Silicalite-2 in Propene Carbonylation to Methyl Butyrate: A DFT Study

**DOI:** 10.3390/molecules30173549

**Published:** 2025-08-29

**Authors:** Lu Wang, Xingyong Wang, Hongchen Li, He Chen, Wanru Feng, Zerun Zhao, Fujun Zhao, Shuai Lei, Zhanggui Hou, Songbao Fu

**Affiliations:** CNOOC Institute of Chemicals & Advanced Materials, Beijing 102209, China

**Keywords:** Rh(II)/Silicalite-2 catalysis, propene, hydroesterification, methyl butyrate, density functional theory

## Abstract

The hydroesterification of olefins provides a highly efficient way to produce high value-added ester products from simple and abundant olefin feedstocks. In this work, DFT calculation was performed to investigate the detailed reaction mechanism of propene hydroesterification over Rh(II)/Silicalite-2 catalysts. Three possible mechanistic pathways were systematically explored and compared in terms of their adsorption configurations, reaction energies, and transition-state barriers. Among them, the Carbonylation-First pathway exhibited the most favorable energy profile with the lowest overall kinetic barriers, indicating it to be the most likely way for ester formation. A comparison of methyl butyrate and methyl isobutyrate formation revealed that the linear product is energetically more favorable, particularly along the Carbonylation-First pathway. Moreover, the Rh(II) center demonstrates a different catalytic effect over conventional Rh(I) species by significantly lowering the energy barrier for CO insertion, a key step in both hydroformylation and hydroesterification. These findings provide fundamental insight into the role of Rh(II)/zeolite systems in carbonylation reactions and offer theoretical guidance for the design of catalysts.

## 1. Introduction

Carbonylation reactions represent a class of fundamental transformations in organometallic catalysis, wherein unsaturated hydrocarbons such as olefins or alkynes react with CO and nucleophiles to generate high value-added oxygenates including aldehydes, acids, esters, and amides [1,2,3,4,5,6,7,8]. These reactions form the basis for numerous industrial processes due to their atom economy and function in constructing C-C and C–heteroatom bonds. Among them, hydroformylation [9,10,11,12,13,14,15,16] and hydroesterification [17,18,19,20,21,22,23,24,25] have attracted widely attention, particularly in the sustainable chemical synthesis and C1 chemistry. In recent years, hydroesterification reactions have emerged as a pivotal route for the direct and selective synthesis of alkyl esters, which integrate olefins, carbon monoxide, and alcohols to produce esters [18,20,25,26,27,28,29]. These esters serve as key intermediates and solvents in the pharmaceutical, fragrance, and polymer industries [30,31]. Compared to traditional esterification of acids and alcohols, hydroesterification offers a more efficient strategy by directly building the carbon skeleton from simple feedstocks. Under this background, the catalytic carbonylation of propene offers an effective route to produce methyl butyrate, a high value-added and widely applicable ester [32,33]. This approach not only enhances the economic value of propene but also simplifies the synthetic process by avoiding the multi-step hydroformylation–oxidation–esterification sequence, thereby providing a promising pathway for the high value-added utilization of abundant propene resources.

Rhodium-based catalysts have long been recognized for their exceptional performance in carbonylation reactions due to their high activity, selectivity, and ability to accommodate diverse ligand environments [34,35,36,37]. In both homogeneous and heterogeneous systems, Rh species exhibit good reactivity to C-C bond formation via the insertion of CO into Rh–alkyl or Rh–hydride intermediates [38,39,40,41,42,43]. While Rh(I) complexes are the most widely studied in traditional carbonylation chemistry, Rh(II) species have recently attracted growing interest owing to their unique electronic structures, redox flexibility, and potential to stabilize unconventional intermediates [44,45,46,47]. In particular, Rh(II) dimers or atomically dispersed Rh(II) centers supported on porous materials have demonstrated promising catalytic activity in various C1 transformations, including oxidative carbonylation, alkoxy carbonylation, and C-H activation. Rh(II) can participate in redox-neutral catalytic cycles, as well as facilitate concerted or cooperative reaction pathways, which offer a new method to design highly efficient carbonylation catalysts [48]. Although Rh(II) species possess favorable catalytic properties, systematic investigations of their catalytic functions remain scarce. This is due to high sensitivity to redox changes and the stringent conditions required to stabilize Rh(II) species. These challenges have hindered their widespread application and limited the development of Rh(II) catalytic systems. In particular, the role of Rh(II) in hydroesterification has been poorly explored, and the mechanistic understanding of its behavior in such a process remains incomplete. Therefore, theoretical studies to elucidate the reaction pathways of Rh(II)-based catalysts have great significance.

Zeolite materials have been extensively employed as catalyst supports due to their high surface area, well-defined pore structures, and tunable framework compositions [21,49,50,51,52]. They can stabilize active metal centers within microporous environments, making them particularly attractive for structure-sensitive reactions. In heterogeneous catalysis, the physicochemical properties of the zeolite framework can significantly influence the activity and selectivity of catalysts. Siliceous (Al-free) zeolites offer distinct advantages for catalytic systems where acidity may induce undesired side reactions [53,54]. In particular, the absence of framework aluminum eliminates Brønsted acid sites, thereby suppressing acid-catalyzed pathways such as the formation of C^+^ species. This is especially critical in hydroesterification reactions. The presence of acid sites can lead to C^+^ species side reactions, resulting in multiple by-products and reduced selectivity. In this context, a MEL-type zeolite Silicalite-2 has emerged as a promising support due to its three-dimensional pore system composed of elliptical 10-membered ring channels, which provides excellent molecular confinement and diffusional properties [55,56]. Notably, a recent pioneering study demonstrated that Silicalite-2 zeolite exhibits significant advantages in shape-selective catalysis, achieving a 99% regioselectivity for *n*-butanal in the hydroformylation of propene on the Rh/MEL catalyst [57,58]. Therefore, these characteristics make Silicalite-2 an ideal support for studying Rh(II)-catalyzed hydroesterification reactions.

Building on the above considerations, the objective of this study is to provide a detailed theoretical investigation of the reaction mechanism of Rh(II)/Silicalite-2-catalyzed hydroesterification of propene with CO and methanol. Utilizing density functional theory (DFT), we aim to explore the elementary steps involved in the formation of methyl butyrate, including the adsorption of reactants, the CO insertion process, and the subsequent esterification step. Aiming at the development of highly efficient and sustainable catalysts for hydroesterification reactions, providing valuable guidance for the design of catalysts.

## 2. Results and Discussion

### 2.1. Adsorption Test

**Figure 1:**
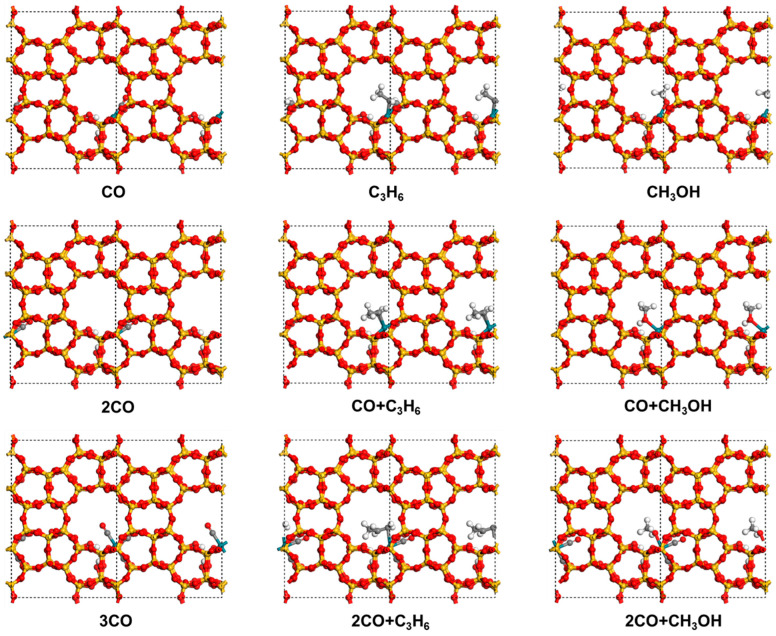
Adsorption structures of CO, C_3_H_6_, and CH_3_OH on Rh(II)/Silicalite-2 under single-, double-, and triple-molecule co-adsorption. C atoms are shown in gray.

The adsorption of CO, C_3_H_6_, and CH_3_OH on Rh(II)/Silicalite-2 was investigated. As summarized in Table 1 and illustrated in Figure 1, when each molecule was adsorbed at the Rh site, CO exhibited the strongest adsorption affinity. It bonded to the Rh via its C end with an adsorption energy of −2.12 eV. In comparison, C_3_H_6_ interacted with the Rh through its C=C double bond with an adsorption energy of −1.64 eV. CH_3_OH bonded to the Rh through the O with a lower adsorption energy of −1.50 eV. These results indicate that CO with a stronger electron-withdrawing ability forms a more stable coordination with Rh(II) than C_3_H_6_ and CH_3_OH. Therefore, further co-adsorption studies were carried out based on the most favorable CO adsorption configuration, which provides the largest adsorption energy and serves as a representative intermediate structure for subsequent exploration.

When the second molecule was adsorbed at the Rh site, the relative adsorption strengths followed the same trend as observed for the first molecule adsorption. CO again exhibited the strongest interaction with an adsorption energy of −1.66 eV, followed by C_3_H_6_ with −1.42 eV, and CH_3_OH with −1.22 eV. Based on these results, the dual-CO adsorption configuration was selected as the platform for exploring the adsorption behavior of the third molecule. Interestingly, upon the adsorption of the third molecule at the Rh site, the adsorption energies of CO, C_3_H_6_, and CH_3_OH converged in −1.00 eV. This phenomenon may be attributed to the strong π-accepting nature of CO, which substantially depleted the electron density of the Rh sites upon dual CO coordination. As a result, the Rh center’s electron-donating ability was reduced, leading to diminished adsorption strengths for all three third adsorption molecule candidates.

Although the CO + CO + CH_3_OH configuration shows a slight advantage in both total adsorption energy and the third molecule adsorption energy, the CO + CO + CO structure was chosen as the initial state for subsequent mechanistic investigations. This selection allows for a more comprehensive examination of the overall reaction, as it can evolve into alternative intermediate states such as CO + CO + CH_3_OH and CO + CO + C_3_H_6_ during the catalytic cycle.

### 2.2. The Reaction Mechanism of Hydroesterification of Propene

#### 2.2.1. Hydrogenation-First Pathway for Methyl Butyrate and Methyl Isobutyrate Formation

**Figure 2:**
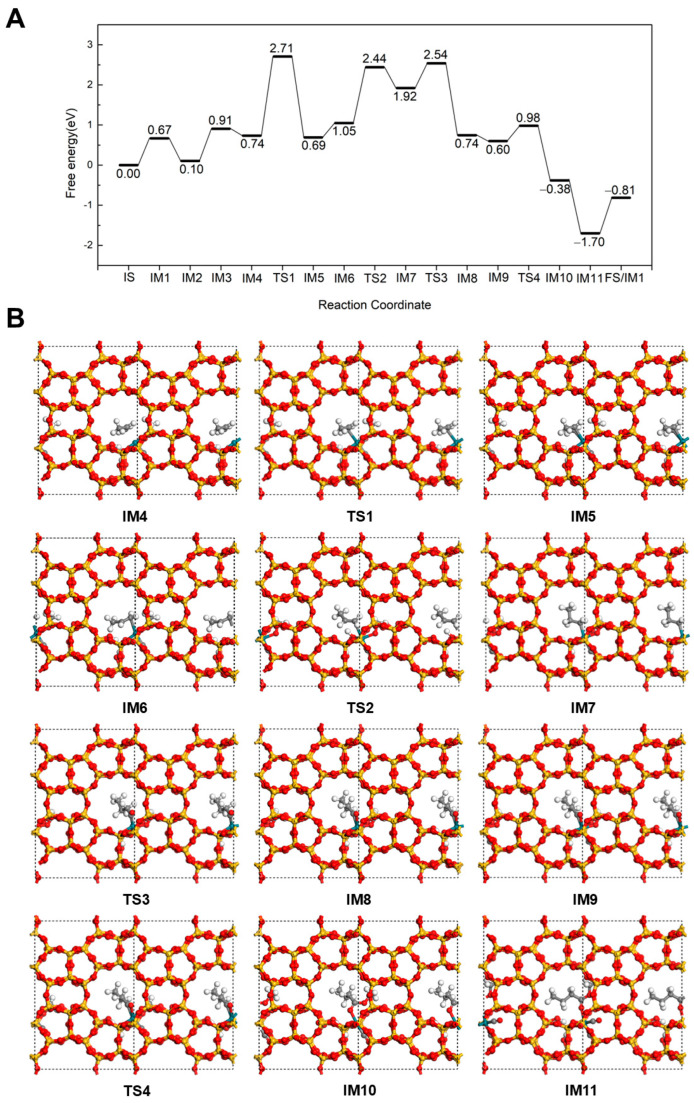
The energy profile (**A**) and illustrated mechanism (**B**) of the Hydrogenation-First pathway for methyl butyrate formation.

We investigated a reaction mechanism similar to the hydroformylation pathway of propene, wherein propene first undergoes hydrogenation to form a propyl intermediate, followed by CO insertion to generate an acyl species, and finally reacts with a methoxy group to yield methyl butyrate at 298 K. As shown in Figure 2, the reaction initiates from the co-adsorption structure containing three CO molecules, denoted as IS (3CO in Figure 1). One CO molecule desorbs from the Rh(II) after overcoming a barrier of 0.67 eV, leading to intermediate IM1 (2CO in Figure 1). Subsequently, a propene molecule adsorbs at the Rh site via its C=C double bond with −0.57 eV of energy to form the intermediate labeled 2CO + C_3_H_6_ in Figure 1. Another CO molecule desorbs from the Rh with a 0.81 eV energy barrier (CO + C_3_H_6_ in Figure 1). CH_3_OH then adsorbs via its O at Rh with a 0.17 eV of energy release (IM4). Next, the H transfers from the OH group of CH_3_OH to the framework O of the Silicalite-2 zeolite (IM5). This step is slightly exothermic with 0.05 eV, but it needs to overcome a large barrier of 1.97 eV (TS1). To facilitate H coupling, the adsorbed C_3_H_6_ structure undergoes reorientation, positioning the internal C closer to the dissociated H (IM6) with a 0.36 eV energy adsorption. This is followed by the H addition to the internal C to form a *n*-propyl intermediate, which coordinates to the Rh (IM7) via a terminal unsaturated C. This hydrogenation step is also endothermic (ΔE = 0.87 eV) and requires a transition-state barrier of 1.39 eV (TS2). As the reaction proceeds, the *n*-propyl group couples with an adjacent CO molecule adsorbed on Rh to form a *n*-butyryl intermediate (IM8). This key step has a low energy barrier of 0.62 eV (TS3) and 1.18 eV reaction energy. Then, an additional CO adsorbs at the vacant Rh site with the 0.14 eV adsorption energy (IM9). Next, the OCH_3_ on Rh attacks the acyl intermediate to form methyl butyrate (IM10), which binds to the Rh through its carbonyl O. This step proceeds via a low energy barrier of 0.38 eV (TS4). The formation of the ester occurs with a reaction energy of −0.98 eV. A new CO molecule then replenishes the Rh site, releasing 1.32 eV of energy. Finally, the methyl butyrate desorbs from the Rh with a desorption energy of 0.89 eV (FS), thereby completing the catalytic cycle and regenerating the structure corresponding to IM1 for the next cycle.

**Figure 3:**
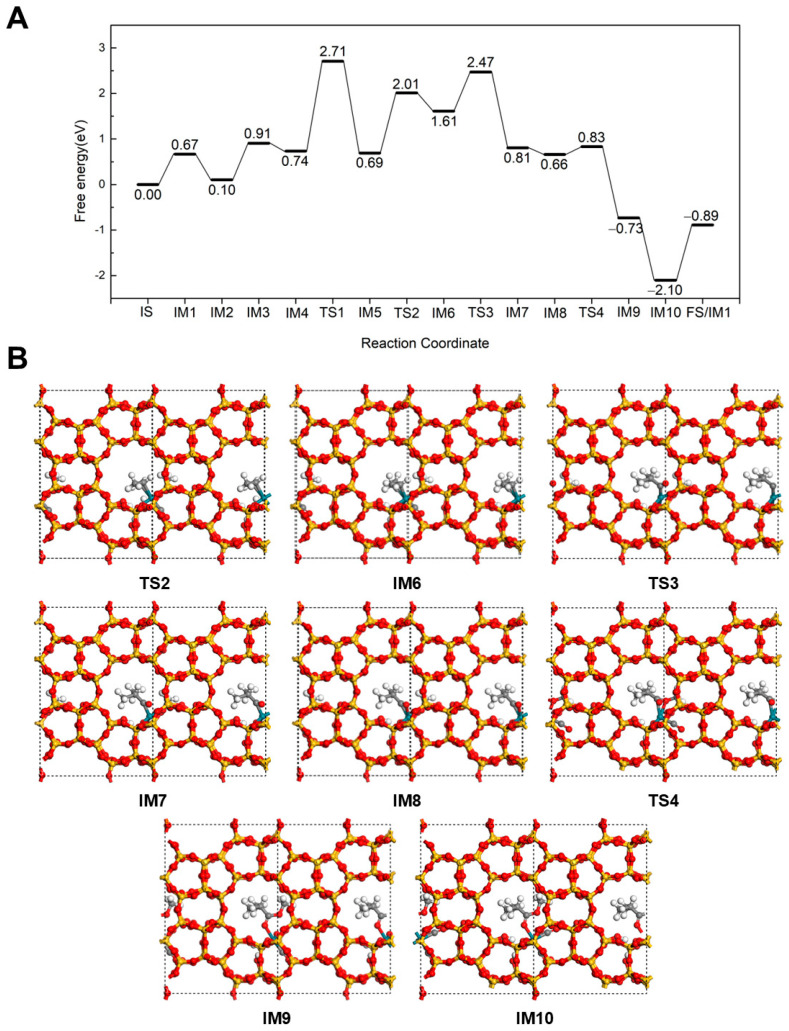
The energy profile (**A**) and illustrated mechanism (**B**) of the Hydrogenation-First pathway for methyl isobutyrate formation.

The reaction mechanism for the formation of methyl isobutyrate at 298 K is illustrated in Figure 3. Up to intermediate IM5, the reaction pathway is very similar to that of methyl butyrate formation. The difference begins with the hydrogenation of C_3_H_6_ to form an isopropyl intermediate. In this pathway, the terminal C of the C=C bond in C_3_H_6_ couples with the dissociated H to create an isopropyl, which binds to the Rh via the central C, resulting in IM6. This hydrogenation step proceeds via TS2 with an energy barrier of 1.32 eV and a reaction energy of 0.92 eV. Subsequently, a CO inserts into the Rh-C bond of the isopropyl to form an *iso*-butyryl species (IM7) with a −0.80 eV reaction energy after overcoming a barrier of 0.86 eV (TS3). The newly created adsorption site on Rh is quickly filled by a CO with an energy release of 0.15 eV. Next, the *iso*-butyryl group reacts with the adsorbed OCH_3_, forming methyl isobutyrate (IM9) via coupling at the carbonyl carbon. This step is exothermic by 1.39 eV and proceeds with a low energy barrier of just 0.17 eV (TS4), indicating a kinetically favorable esterification step. Similar to the linear ester case, the product binds to the Rh through its carbonyl O. A new CO molecule then binds to the Rh site with a 1.37 eV energy release (IM10). Finally, methyl isobutyrate desorbs from the Rh by overcoming a desorption barrier of 1.21 eV, completing the catalytic cycle.

A comparison of the key steps in the reaction pathways leading to methyl butyrate and methyl isobutyrate reveals that both routes exhibit similar energy profiles. In the hydrogenation step, the two pathways show nearly identical behavior, with the energy barriers differing by less than 0.10 eV. In the CO insertion step, the linear pathway toward methyl butyrate is more favorable, displaying a 0.24 eV lower barrier compared to the branched route. Conversely, the branched pathway toward methyl isobutyrate exhibits an advantage in the esterification step, with its barrier being 0.21 eV lower than that of the linear pathway. Given these subtle energy differences in opposite directions, it is difficult to determine which pathway is more favorable overall conclusively.

#### 2.2.2. Methoxycarbonyl-Driven Pathway for Methyl Butyrate and Methyl Isobutyrate Formation

**Figure 4:**
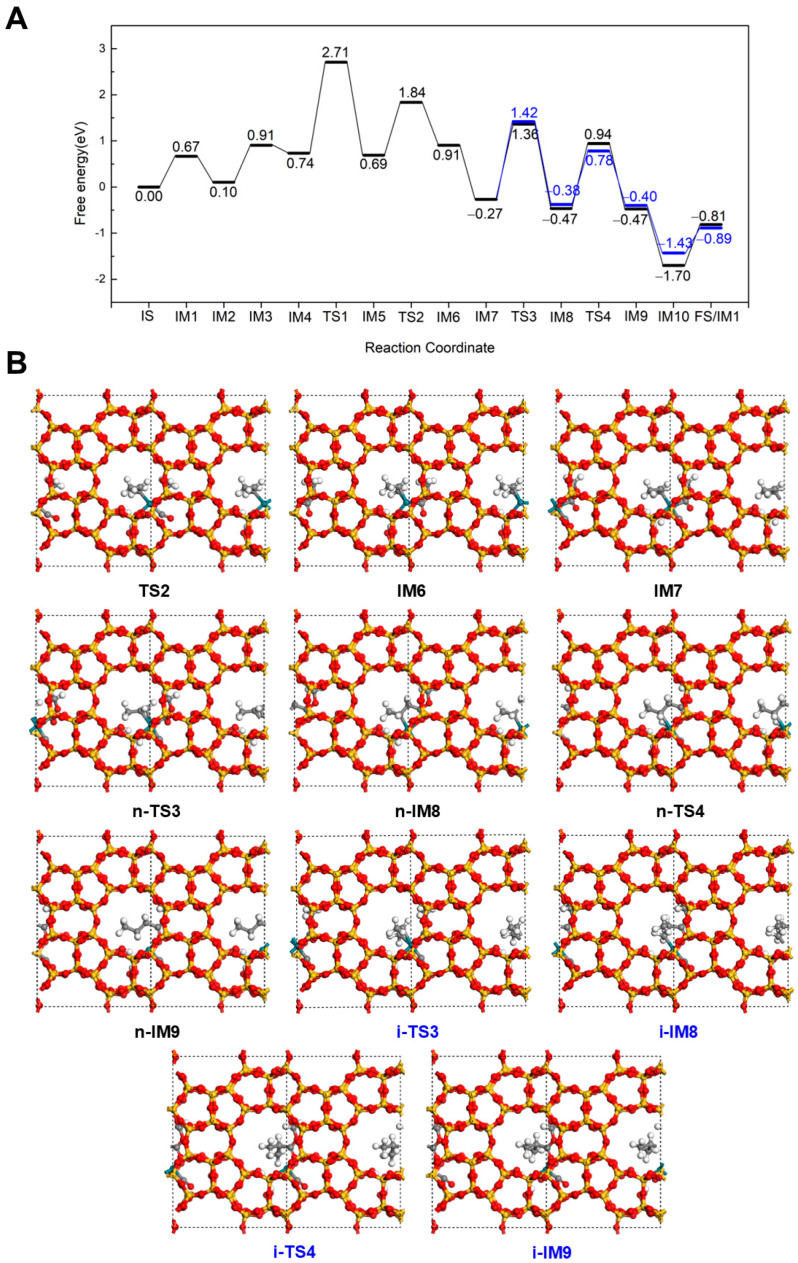
The energy profile (**A**) and illustrated mechanism (**B**) of the Methoxycarbonyl-Driven pathway for methyl butyrate (Black) and methyl isobutyrate formation (Blue).

As shown in Figure 4, an alternative pathway for the formation of methyl butyrate at 298 K was explored, which is different from the conventional hydroformylation-like pathway. In this pathway, all steps up to IM5 are similar to those in the Hydrogenation-First pathway sequence. However, the reaction proceeds via a Methoxycarbonyl-Driven pathway from IM5. In this mechanism, the adsorbed CO interacts with the dissociated OCH_3_ group via TS2, forming a COOCH_3_ intermediate that connects to Rh through the carbonyl C (IM6). This step requires overcoming an energy barrier of 1.15 eV and is slightly endothermic with a reaction energy of 0.22 eV. Subsequently, a new CO rapidly adsorbs at the Rh site, releasing 1.18 eV of energy. Next, the COOCH_3_ group reacts with the terminal C of C_3_H_6_, leading to the formation of an intermediate CH_3_CHCH_2_COOCH_3_ (n-IM8) that continues along the linear methyl butyrate pathway. This step proceeds via n-TS3 with an energy barrier of 1.63 eV and an exothermic reaction energy of 0.20 eV. The resulting intermediate then undergoes hydrogenation with the dissociated H to form methyl butyrate, which binds to Rh via the carbonyl O (n-IM9). This final transformation step via n-TS4 with an energy barrier of 1.41 eV and a reaction energy of 0.06 eV. The subsequent steps are similar to those in Hydrogenation-First pathway in Figure 2. A CO molecule adsorbs to Rh with an adsorption energy of −1.23 eV, followed by desorption of the product methyl butyrate with a desorption energy of 0.89 eV, thereby completing the catalytic cycle. Alternatively, when the COOCH_3_ group reacts with the internal C of C_3_H_6_, the branched intermediate CH_3_CH(COOCH_3_)CH_2_ (i-IM8) is formed. This step occurs via i-TS3 with an energy barrier of 1.69 eV and a reaction energy of −0.11 eV. The intermediate then undergoes hydrogenation via i-TS4 to yield methyl isobutyrate (i-IM9), with a barrier of 1.16 eV. The remaining steps are also similar to those described in Hydrogenation-First pathway for the isobutyrate pathway (Figure 3). A comparison of the two Methoxycarbonyl-Driven pathways shows that the overall energy profiles are largely similar. No clear energetic and kinetic preference is observed between the two pathways.

#### 2.2.3. Carbonylation-First Pathway for Methyl Butyrate and Methyl Isobutyrate Formation

**Figure 5:**
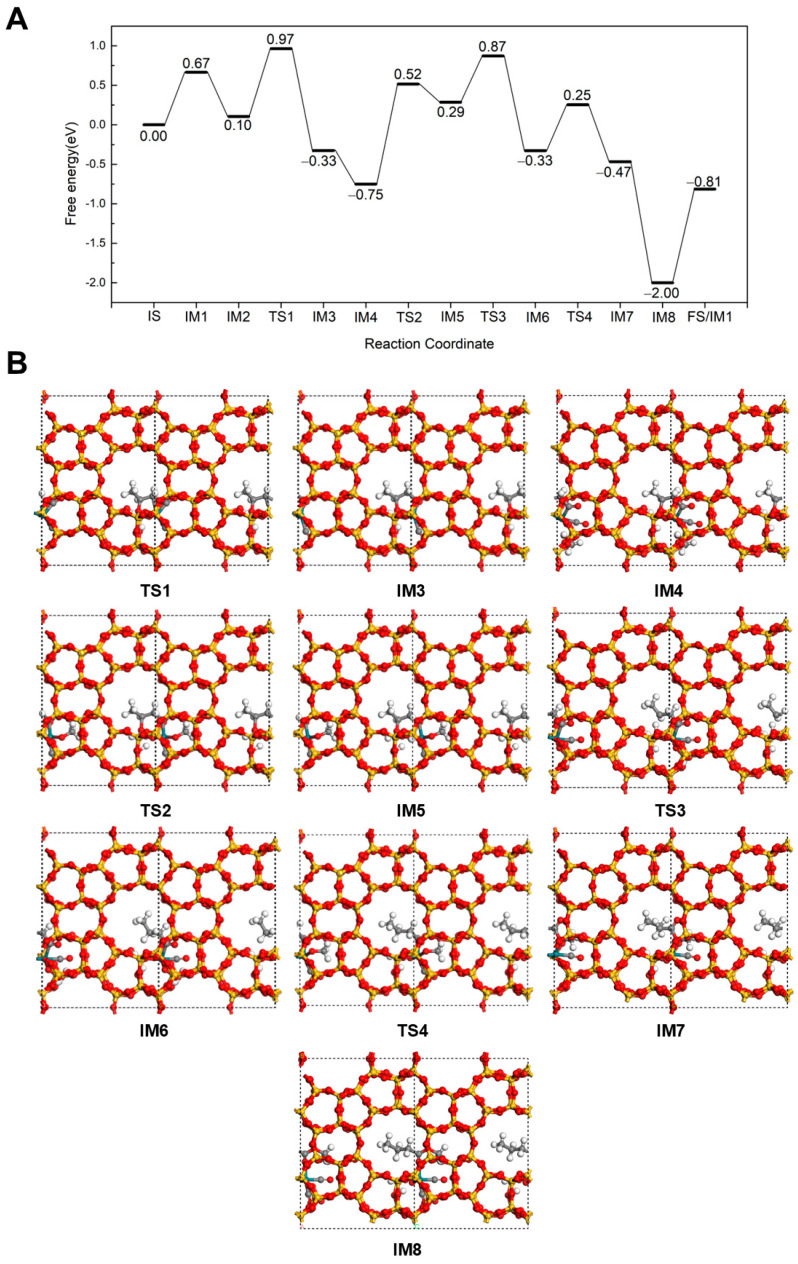
The energy profile (**A**) and illustrated mechanism (**B**) of the Carbonylation-First pathway for methyl butyrate formation.

A novel reaction pathway was further investigated at 298 K, in which CO inserts into propene first, followed by hydrogenation to form an acyl intermediate and, finally, esterification to produce methyl butyrate. The mechanistic details for the formation of methyl butyrate via this pathway are illustrated in Figure 5. This pathway differs from those discussed in Sections Hydrogenation-First pathway and Methoxycarbonyl-Driven pathway, initiating after the adsorption of C_3_H_6_ on Rh (IM2). In the first step, C_3_H_6_ directly reacts with CO to form the CH_3_CHCH_2_CO intermediate (IM3), proceeding through a transition state (TS1) with an energy barrier of 0.87 eV and an exothermic energy of 0.43 eV. CH_3_CHCH_2_CO intermediate adopts a unique structure on the Rh/Silicalite-2 catalyst, in which one end is adsorbed on Rh through the carbonyl C, and the other end is connected to the framework O of zeolite through the unsaturated C in -CH-. A CH_3_OH then occupies the Rh site with releasing 0.42 eV of energy (IM4). Subsequently, after overcoming an energy barrier of 1.27 eV (TS2), the H from the hydroxyl group of CH_3_OH dissociates and transfers to a framework O, yielding IM5 with a reaction energy of 1.04 eV. Similar to other pathways in 3.2.1 and 3.2.2, the dissociation of H from CH_3_OH requires a relatively high energy barrier and is strongly endothermic. Next, the dissociated H transfers to the -CH- group via a transition state (TS3) with a barrier of 0.58 eV, releasing 0.62 eV of reaction energy. The CH_3_CHCH_2_CO intermediate converts into a n-butyryl species adsorbed on Rh (IM6) and breaks the bond between the saturated C and the zeolite O at the same time. Next, the OCH_3_ reacts with the *n*-butyryl intermediate to form methyl butyrate (IM7). This esterification step proceeds via TS4 with an energy barrier of 0.58 eV and an exothermic energy of 0.14 eV. Finally, a CO molecule replenishes the Rh site (IM8), followed by desorption of methyl butyrate (IM9). Thereby, the catalyst is recovered to the initial IM1 state for the next catalytic cycle.

**Figure 6:**
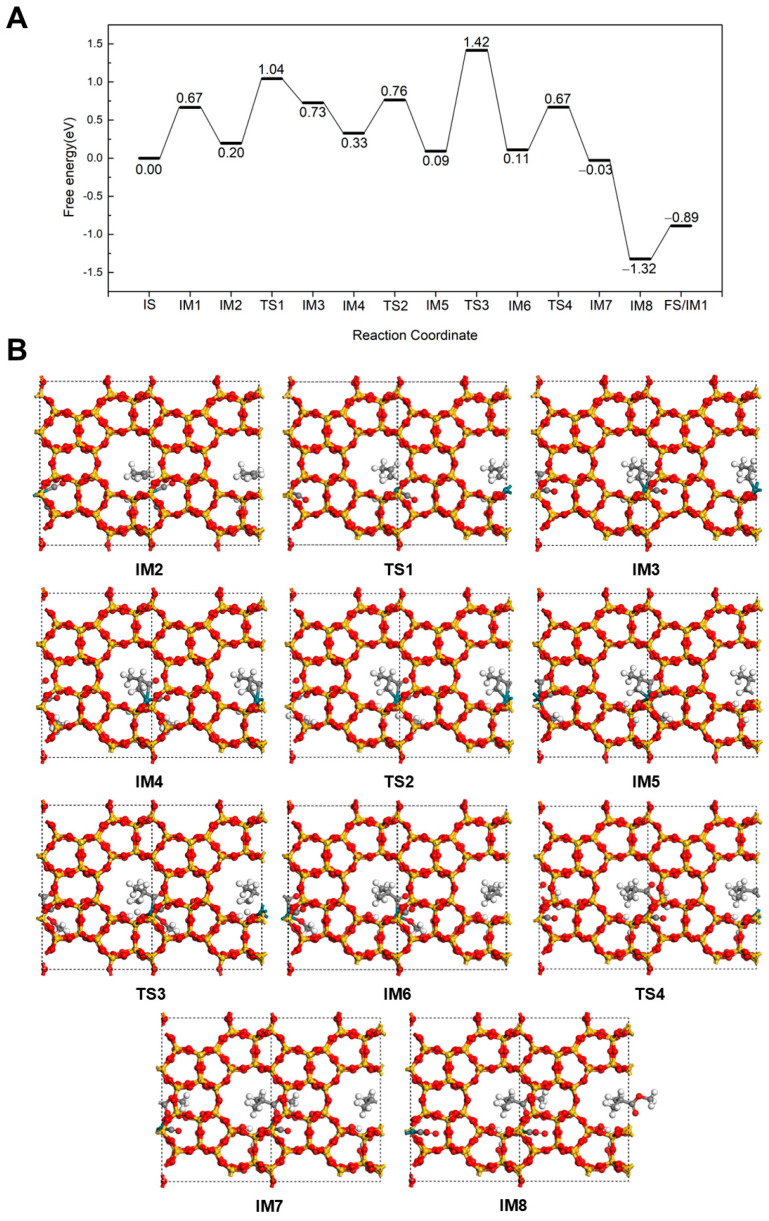
The energy profile (**A**) and illustrated mechanism (**B**) of the Carbonylation-First pathway for methyl isobutyrate formation.

In the methyl isobutyrate formation pathway (Figure 6), C_3_H_6_ adsorbs at the Rh via the C=C bond in a favorable configuration for reaction (IM2). The adsorption energy is calculated to be −0.47 eV, which is comparable to the −0.57 eV adsorption energy of the alternative configuration, indicating that this adsorption structure is also stable. Then, C_3_H_6_ reacts with CO to form the intermediate CH_3_CH(CO)CH_2_ (IM3) via a transition state (TS1) with an energy barrier of 0.84 eV, accompanied by an endothermic reaction energy of 0.53 eV. In this intermediate, the newly formed carbonyl group coordinates to Rh, while the terminal unsaturated carbon continues interacting with the Rh site. Subsequently, CH_3_OH adsorbs at Rh with an adsorption energy of −0.40 eV (IM4). After overcoming a barrier of 0.43 eV (TS2), H dissociates from the OH of CH_3_OH (IM5). The dissociated H then attaches to the CH_3_CH(CO)CH_2_ intermediate, forming the isobutyryl species (IM6) by overcoming a transition state barrier of 1.33 eV (TS3). At this stage, the acyl intermediate coordinates to Rh only through the carbonyl group with the terminal C saturated. Next, the isobutyryl species reacts with the OCH_3_ to form methyl isobutyrate (IM7). This esterification step proceeds with an energy barrier of 0.56 eV (TS4) and an exothermic energy of 0.14 eV. Finally, as a CO adsorbs at the Rh site, methyl isobutyrate desorbs from the catalyst by overcoming an energy barrier of 0.43 eV. The highest energy barriers in both methyl butyrate and methyl isobutyrate pathways are similar, indicating that neither pathway exhibits a distinct advantage.

### 2.3. Discussion

We have summarized the energy barriers of four key transition states for the various reaction pathways from Section 2.2 in Table 2. A comparative analysis of these critical steps across the three main pathways, including both the linear (methyl butyrate) and branched (methyl isobutyrate) pathways, indicates no clear dominance in the selectivity toward either product from these four steps. However, the Methoxycarbonyl-Driven Pathway (Section 2.2.2) appears to be the least competitive among the pathways examined, as all four transition state energy barriers exceed 1.00 eV for both the linear and branched products, significantly increasing the reaction difficulty. Notably, the H dissociation from CH_3_OH in this pathway has an energy barrier almost 2.00 eV. A similar issue exists in the Hydrogenation-First Pathway (Section 2.2.1), which also poses a high energy barrier of 1.97 eV for hydrogen dissociation. In addition to a CO insertion barrier exceeding 1.00 eV, thereby limiting its overall competitiveness. In contrast, the Carbonylation-First Pathway (Section 2.2.3) demonstrates good competitiveness with only one transition state barrier exceeding 1.00 eV in each of the linear and branched product pathways. The highest barrier in this pathway is approximately 1.30 eV, much lower than the 1.97 eV maximum barriers in the Hydrogenation-First pathway and Methoxycarbonyl-Driven pathway. Therefore, it can be seen that the Carbonylation-First pathway is the most favorable pathway for Rh(II)/Silicalite-2 catalyzed propene carbonylative esterification to methyl butyrate.

By comparing the energy barriers of the four key transition states in the Carbonylation-First pathway for methyl butyrate and methyl isobutyrate formation, the most significant difference is the H dissociation from CH_3_OH and the subsequent H transfer to the propylene derivatives intermediate. In the methyl butyrate pathway, the barriers for CH_3_OH dissociation and H addition to the propene intermediate are 1.27 eV and 0.58 eV, respectively. This trend in energy barrier size is consistent with those observed in the Hydrogenation-First and Methoxycarbonyl-Driven pathways. However, in the methyl isobutyrate pathway, this trend is reversed. The CH_3_OH dissociation barrier is much lower at 0.43 eV, whereas the barrier for H transfer to the propylene derivatives intermediate increases to 1.33 eV. This behavior may be attributed to the adsorption structure of the CH_3_CH(CO)CH_2_ intermediate (IM3), which binds to Rh via both the carbonyl C and the unsaturated C. The interaction between Rh and the zeolite framework O weakens when CH_3_OH is at Rh, making the framework O more receptive to H and thereby significantly lowering the CH_3_OH dissociation barrier. Conversely, this stronger interaction between H and the framework O results in a higher energy barrier (1.33 eV) for H migration.

A more detailed comparison between the linear and branched pathways within the Carbonylation-First mechanism reveals a distinct energetic preference for methyl butyrate formation. It can be found that, if the reaction begins from the co-adsorption of 2 CO and C_3_H_6_ (IM2), the highest-energy transition state for methyl butyrate is 0.87 eV, significantly lower than the corresponding 1.22 eV required for methyl isobutyrate. Moreover, the secondary highest transition state along the linear pathway is 0.77 eV, compared to 0.84 eV for the branch pathway. This indicates that the formation of methyl butyrate follows a smoother energy landscape, resulting in a thermodynamically and kinetically more favorable transformation.

The structural properties of the Silicalite-2 support further enhance this selectivity. Silicalite-2 imposes shape constraints that disfavor bulky branched intermediates while stabilizing linear transition states. Such a confinement effect is known to contribute to regioselectivity in olefin transformations. To substantiate this interpretation, we analyzed the pore-fit metrics of the transition states (Table 3). The branched transition states exhibit consistently smaller wall clearances (*d*_min_ = 1.86–2.14 Å, ⟨*d*_3_⟩ = 2.01–2.29 Å) than the linear counterparts (*d*_min_ = 2.28–2.56 Å, ⟨*d*_3_⟩ = 2.45–2.65 Å), confirming that steric crowding penalizes the branched pathway inside the Silicalite-2 channels. Therefore, both energetic and geometric analyses point to a clear preference for methyl butyrate formation, consistent with the experimentally observed selectivity of Rh/Silicalite-2 catalysts toward n-butyraldehyde [57].

In the carbonylation of propene, both hydroformylation and hydroesterification involve two key steps: the hydrogen addition to propene or its derivatives and the CO insertion. We have summarized the energy barriers in Table 4 for these two steps in the Rh(II) catalytic system from pathways 3.2.3 and 3.2.1, alongside corresponding barriers reported for Rh(I)-catalyzed propene hydroformylation. The comparison reveals that Rh(I) systems have a clear advantage in the hydrogen addition step to propene, with energy barriers consistently below 0.50 eV. In contrast, Rh(II) exhibits a significant advantage in the CO insertion step, which is usually the rate-determining step in carbonylation. The reaction barrier is lowered by about 0.50 eV compared to that in Rh(I). This may be since the strong electron-withdrawing CO strongly binds to the high electron density Rh(I) center, thus inhibiting the CO participation in the reaction. Similarly, electron-donating H also experiences a relatively higher energy barrier in the low electron density Rh(II) environment.

It should be noted that the present study employs a periodic, gas-phase DFT model of Rh(II)/Silicalite-2 to elucidate intrinsic reaction pathways and electronic effects in a well-defined, confined environment. While this approach reliably captures local geometric and electronic interactions that determine key steps, it does not explicitly treat solvent molecules or the effect of high gas-phase pressures that are relevant to practical hydroesterification. Reliable implicit solvation models for fully periodic microporous frameworks remain challenging, and explicit solvent treatments would require much larger supercells and extensive sampling by AIMD, which are beyond the computational scope of the present work. Quantitative predictions of rates and selectivities under realistic liquid-phase or high-pressure conditions will require advanced extensions such as (1) explicit solvent/AIMD sampling or QM/MM approaches to capture solvation and dynamic effects, (2) grand-canonical or microkinetic treatments to include pressure/coverage, and (3) targeted experimental validation.

## 3. Calculation Method

All calculations were performed using DFT within the plane-wave pseudopotential as implemented in the VASP code [59,60] with a cutoff energy of 400 eV [57,58]. The exchange and correlation potential was described with the Perdew–Burke–Ernzerhof (PBE) of the generalized gradient approximation (GGA) [61]. Van der Waals correction (D3) was applied in all calculations. The convergence threshold for self-consistent-field iteration was set to 10^−4^ eV. To locate transition states within the system, the Climbing Image Nudged Elastic Band (CI-NEB) method was employed [62,63]. The atomic positions were relaxed until the force on each atom was less than 0.05 eV/Å. The averaged electrostatic potential in the planes perpendicular to the slab normal could be obtained using the periodic slab model and self-consistent dipole correction. Γ-Centered k-point meshes of 1 × 1 × 1 is used for the Brillouin zone integration. The free energy was corrected at 298 K according to the method in the literature [64].

**Figure 7:**
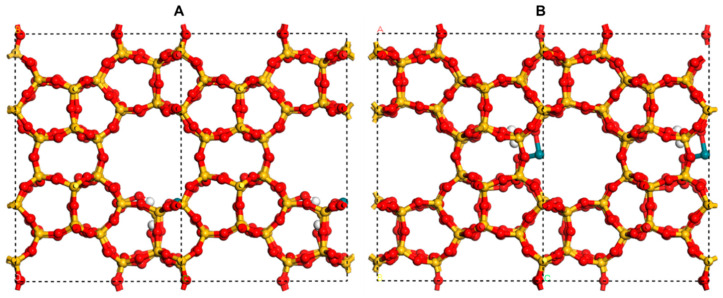
(**A**) Side view and (**B**) top view of the model of Rh(II)/Silicalite-2. Si, O, H, and Rh atoms are shown in wheat, red, white, and dark green, respectively.

The bulk model [RhH(CO)_2_]@MEL as reported in the literature [57] was employed, a zeolite characterized by its MEL structure with Rh(I). The parameters for the unit cell are a = b = 20.33 Å and c = 13.59 Å. They substituted one Si atom for the Si initially occupying the MEL channel. To compensate for the charge imbalance created by the Rh substitutions, H atoms are introduced into the system. Based on the model, all adsorption species (CO and H) at the Rh site are removed. Then the model with Rh(II) for this research (Figure 7).

## 4. Conclusions

In this work, DFT was employed to explore the hydroesterification mechanism of propene over Rh(II)/Silicalite-2 catalysts. Hydrogenation-First, Methoxycarbonyl-Driven, and Carbonylation-First, three possible reaction pathways, were systematically compared. Among them, the Carbonylation-First pathway demonstrated the lowest overall energy barriers and the most favorable kinetic profile, making it the most competitive pathway for methyl butyrate formation.

A detailed comparison between the formation of methyl butyrate and methyl isobutyrate revealed that the linear product is energetically more favorable, especially in the Carbonylation-First pathway. This selectivity originates from more balanced hydrogen activation and transfer steps, as well as a smoother overall energy landscape. These results suggest that Rh(II)/Silicalite-2 catalysts intrinsically favor n-butyrate formation during hydroesterification, consistent with prior observations in hydroformylation reactions. In addition, the confined pore environment of the Silicalite-2 zeolite provides a shape-selective effect that sterically disfavors bulky branched intermediates, thereby further enhancing the selectivity toward linear ester products.

This study demonstrates that Rh(II)/Silicalite-2 catalysts exhibit unique mechanistic characteristics distinct from Rh(I) systems. While Rh(I) catalysts are known for their efficient hydrogenation steps, our analysis reveals that Rh(II) catalysts possess a clear advantage in facilitating CO insertion, a key step in both hydroformylation and hydroesterification. These results provide a theoretical basis for their further application in C1 chemistry and olefin valorization.

## Figures and Tables

**Table 1:** Adsorption energies (eV) of CO, C_3_H_6_, and CH_3_OH on Rh(II)/Silicalite-2 under single-, double-, and triple-molecule co-adsorption at 298 K.

State	Adsorption Molecular	Total Adsorption Energy	Adsorption Energy
1st molecular adsorption	CO	−2.12	−2.12
	CH_3_OH	−1.50	−1.50
	C_3_H_6_	−1.64	−1.64
2nd molecular adsorption based on CO adsorption	CO + C_3_H_6_	−3.53	−1.42
	2CO	−3.77	−1.66
	CO + CH_3_OH	−3.34	−1.22
3rd molecular adsorption based on 2CO adsorption	3CO	−4.40	−0.67
	2CO + CH_3_OH	−4.74	−0.96
	2CO + C_3_H_6_	−4.35	−0.57

**Table 2:** Energy barriers (eV) of key transition states in different reaction pathways for methyl butyrate and methyl isobutyrate formation.

Pathway	Methyl Butyrate	The Dissociation of H from CH_3_OH	H Combines with Propylene Derivatives	CO Insertion	OCH_3_/COOCH_3_ Combines with Propylene Derivatives
Hydrogenation-First	*n*	1.97	0.62	1.39	0.38
	*iso*	1.97	0.86	1.32	0.17
Methoxycarbonyl-Driven	*n*	1.97	1.63	1.15	1.41
	*iso*	1.97	1.69	1.15	1.16
Carbonylation-First	*n*	1.27	0.58	0.87	0.58
	*iso*	0.43	1.33	0.84	0.56

**Table 3:** Geometric analysis of Carbonylation-First pathway transition states in Silicalite-2.

Transition State	*d*_min_/Å	⟨*d*_3_⟩/Å
*n*-TS1	2.31	2.45
*n*-TS2	2.56	2.65
*n*-TS3	2.47	2.55
*n*-TS4	2.28	2.48
*iso*-TS1	1.96	2.01
*iso*-TS2	2.09	2.22
*iso*-TS3	1.86	2.09
*iso*-TS4	2.14	2.29

**Table 4:** Energy barriers (eV) for Hydrogenation and CO Insertion steps in propene carbonylation reaction by Rh(II) system and reported Rh(I) systems [57].

Reaction Pathway	Line or Branch Routs	The Combination of H	CO Insertion
Carbonylation-First in Rh(II)	*n*	0.58	0.87
	*iso*	1.33	0.84
Hydrogenation-First in Rh(II)	*n*	0.62	1.39
	*iso*	0.86	1.32
Hydroformylation in Rh(I)	*n*	0.15	1.38
	*iso*	0.49	1.80

## Data Availability

The data are contained within the article.
